# Recombination in HIV: an important viral evolutionary strategy.

**DOI:** 10.3201/eid0303.970301

**Published:** 1997

**Authors:** D S Burke

**Affiliations:** Walter Reed Army Institute of Research, Rockville, MD 20850, USA. dburke@pasteur.hjf.org

## Abstract

Human immunodeficiency virus (HIV) is a diploid virus: each virion carries two complete RNA genomic strands. Homologous recombination can occur when a cell is coinfected with two different but related strains. Naturally occurring recombinant HIV strains have been found in infected patients in regions of the world where multiple genotypic variants cocirculate. One recombinant HIV strain has spread rapidly to millions of persons in Southeast Asia. Recombination is a mechanism whereby high level and multidrug-resistant strains may be generated in individual treated patients. Recombination also poses theoretical problems for the development of a safe HIV vaccine. Certain features of HIV replication, such as syncytium formation and transactivation, may be best understood as components of a sexual reproductive cycle. Recombination may be an important HIV evolutionary strategy.


### Recombination in HIV: An Important Viral Evolutionary Strategy

**Donald S. Burke**
Walter Reed Army Institute of Research, Rockville, Maryland, USA

Human immunodeficiency virus (HIV) is a diploid virus: each virion carries two complete RNA genomic strands. Homologous recombination can occur when a cell is coinfected with two different but related strains. Naturally occurring recombinant HIV strains have been found in infected patients in regions of the world where multiple genotypic variants cocirculate. One recombinant HIV strain has spread rapidly to millions of persons in Southeast Asia. Recombination is a mechanism whereby high level and multidrug-resistant strains may be generated in individual treated patients. Recombination also poses theoretical problems for the development of a safe HIV vaccine. Certain features of HIV replication, such as syncytium formation and transactivation, may be best understood as components of a sexual reproductive cycle. Recombination may be an important HIV evolutionary strategy.

Human immunodeficiency virus (HIV)-1, like all retroviruses, is "diploid." Each viral particle contains two RNA strands of positive polarity, each full length and potentially able to replicate (1). No other virus families, RNA or DNA, are diploid. Typically both RNA strands in a retroviral particle derive from the same parent provirus. However, if an infected cell simultaneously harbors two different proviruses, one RNA transcript from each provirus can be encapsidated into a single "heterozygous" virion. When this virion subsequently infects a new cell, the reverse transcriptase may jump back and forth between the two RNA templates so that the newly synthesized retroviral DNA sequence is recombinant between that of the two parents (2). All subsequent progeny virions will be of this recombinant genotype. HIV-1 strains with chimeric genomes thought to have arisen through homologous recombination have recently been discovered in nature (3).

Temin observed that the replication strategy of HIV-1 suggests a form of primitive sexual reproduction (4), which is apparently genderless but sexual in that 1) two parental gametes must fuse into a single progeny, 2) the genetic information of the parental strains is recombined, and 3) subsequent offspring carry genetic information from both parents.

Address for correspondence: Col. Donald S. Burke, Walter Reed Army Institute of Research, 1600 East Gude Drive, Rockville, MD 20850 USA; fax 301-294-1898; e-mail: dburke@pasteur.hjf.org.

### Theoretical Advantages and Disadvantages of Recombination

The replication error rate for HIV is such that each newly synthesized HIV genome carries on average approximately one mutation (5). This high mutation rate, common to most RNA viruses, permits rapid exploration of nucleotide sequence space (the universe of all possible RNA sequences) (6). Only certain regions of sequence space encode replication-competent viruses; these regions can be conceptualized as "peaks" on a "fitness landscape" of sequence space. Although a high mutation rate can lead to rapid evolution, too high a mutation rate carries the danger that the encoded information may degenerate into gibberish. For an organism with a very high mutation rate, an efficient recombination mechanism provides at least two significant theoretical advantages.

### Escape from Muller's Ratchet (Within a Fitness Peak)

For any organism with a genome sequence exactly on a peak on the fitness landscape, every new mutation is by definition not beneficial. Furthermore, unfavorable mutations accumulate more rapidly than restorative back-mutations. Muller showed that in the absence of recombination, the net effect is an inexorable stepwise

"ratcheting down" in fitness of the entire population, where each step in the "ratchet" represents loss of the previously most highly fit genomic sequence. Genetic recombination can readily bring about regeneration of perfectly fit organisms from less than perfectly fit parents (7).

### Evolutionary Broad Jumping (Between Fitness Peaks)

On rugged fitness landscapes—regions of sequence space where as few as one or two mutations can be lethal—an organism may be trapped on a fitness peak because locations in sequence space near the peak may all be nonviable. In such circumstances, step-by-step mutation is not an option for exploration of sequence space. Recombination between two such highly niched organisms can generate progeny that may fortuitously "land" on unexplored fitness peaks at positions in sequence space between those of the parents. Kaufman has dubbed this process "evolutionary broad jumping" (8).

For conventional plus-stranded RNA viruses, replication occurs in the cytoplasm. A plus RNA strand growing on a negative strand template can transiently hybridize with other template (negative) strands to form a replication complex, thereby confusing the polymerase into a "copy choice" that can lead to template switching (9). In contrast, transcription of a retroviral genomic plus strand is restricted to a fixed location in the nucleus where the negative strand template is integrated into the host chromosomal DNA. Unable to utilize the conventional "replication complex" mechanism for recombination during forward transcription, retroviruses may have evolved an alternative mechanism to bring two strands together within a single virion to permit recombination during reverse transcription.

As for all organisms that reproduce sexually, the cost is high: half the genetic information in each generation is wasted. Furthermore, the efficiency of retroviral recombination is unclear. Many "matings" occur between sibling strands derived from the same provirus, which may be identical or differ at only one or two nucleotides. At the other extreme, coinfection of a single cell by two very different lentiviruses may not give rise to any heterozygous virions, and even if copackaging does occur, the degree of sequence identity may be insufficient to permit homologous recombination (10).

### Evidence of HIV Recombination in Nature

In the research laboratory, recombination is widely considered a dominant feature of retroviral genetics. When cell cultures are coinfected with retroviruses that contain genetic markers at specific sites on their genomes, recombinant progeny arise frequently, and markers as close as 1,000 nucleotides segregate "as if unlinked" (1).

The possibility that HIV-1 strains might recombine in nature was proposed early in the epidemic (11). However, the first compelling evidence for lentivirus recombination in nature was the discovery that isolates from sabeus monkeys in western Africa were chimeras between the simian immunodeficiency virus (SIV) of African green monkeys and the SIV of sooty mangabeys (12). The sabeus monkey SIV was shown to be a recombinant resulting from at least two interstrand crossovers between genomes of the green monkey and mangabey viruses.

Most HIV-1 strains from around the world can be placed into one of nine nucleotide sequence-defined clades; these clades have been given the letter designations A through I. However, more than a dozen HIV-1 strains isolated from patients have now been shown to have chimeric genomes in that their gag and env genomic regions cluster with different clades (13). Interclade recombination is relatively easy to demonstrate because strains from different clades typically differ substantially in their nucleotide sequence identities. For example, the env gene sequences of HIV-1 strains of different clades may differ by 20% or more (14). As might be expected, interclade HIV-1 recombinants have most often been detected in geographic regions where two or more clades are prevalent (14). For example, A clade and D clade viruses cocirculate in East Africa, and several A/D recombinant viruses have been detected in this region. In western equatorial Africa, multiple HIV-1 clades (A, C, D, E, F, G, and H) as well as the outlier "group O" HIV-1 strains are known to cocirculate, and preliminary studies suggest that recombinant forms are quite common in this region. Most are recombinants between the A clade, which is predominant, and another clade. The rapidly spreading HIV-1 strain in Southeast Asia is one such recombinant, of A with E (15). This strain consists of A clade gag and pol genes, but the env gene is chimeric: the surface gp120 envelope protein and external domain of transmembrane gp41 envelope protein are contributed by the E clade, while the cytoplasmic domain of gp41 is again A genotype. In effect, this epidemic strain is a pseudotyped A virus that carries an E envelope. A wide variety of genetically similar recombinant E/A strains have been found in equatorial Africa, so it is likely that the recombinant event occurred there and a subclone was introduced into Southeast Asia by an infected traveler. B/F recombinants have been found in Brazil, where both parental clades are found (16).

Intraclade recombinants are much more difficult to detect and demonstrate convincingly because of the genetic similarity of the parental strains. Clones of B clade viruses from the blood of a patient with acute retroviral syndrome who had had multiple sex partners were found to belong to three distinct clade B env variants (17). Some of the clones appeared to be probable recombinants. Strains from an infant who had been transfused with blood from two HIV-infected donors in 1984 were found to include probable B intraclade recombinants (18).

Studies of specimens from an A/C-infected spousal pair in Zambia have shown that a variety of recombinants can be present in one small epidemiologic cluster at different times, suggesting that recombination may be continuous and ongoing in vivo in patients who are coinfected with two or more distinct strains (19).

### Mechanisms of Retroviral Recombination

The exact mechanism by which two retroviral RNA genome strands are copackaged into a single virion—"mating"—is only partially understood. A key step is thought to be the dimerization of the two strands near their 5' genome termini (20), which in turn permits interaction of the RNA packaging signals with gag proteins.

Two genomes per virion is a necessary but not sufficient condition for retroviral recombination: the reverse transcriptase must also readily switch strands (21). Low processivity (loose adherence to the template RNA) is an inherent property of retroviral reverse transcriptases. In the normal retroviral replication cycle, the reverse transcriptase and approximately 1,000 bp of the nascent DNA minus strand jump from the plus RNA strand 5' repeat region to the identical repeat region at the 3' end of the genome. Presumably the low processivity required to permit this jump from one end of the genome to the other also permits ready interstrand switching (4).

Preferred sites for HIV recombination, if any, remain uncertain. If recombination "hot spots" are found, they may be dictated by RNA secondary structures that retard polymerase movement, as with other viruses. Alternatively, physical sites of recombination may be essentially randomly distributed along the genome, and apparent recombination hot spots might simply reflect selection for viability.

### HIV as a Primitive Sexual Organism

Once the replication of HIV is viewed as that of a primitive sexual organism (diploid with mating), it is instructive to reexamine the biology of HIV for other features that might facilitate a sexual life style.

### Syncytium Induction

Some HIV strains induce formation of multinucleated syncytia in cell cultures in vitro; this property has been associated with clinical virulence (22). Syncytia formation might facilitate multiple infection of a single cell by fusing two or more infected cells into one. Syncytium-inducing strains may be more virulent not because syncytium induction per se leads directly to immunopathogenesis, but because this property permits more efficient generation of rapidly growing variants through recombination and selection. Syncytia induction might, therefore, represent a mechanism to optimize the spatial interactions between strains: a mating ground.

### tat/tar Transactivation

Integrated HIV provirus remains transcriptionally inactive unless the LTR promoter region is activated by cellular activation factors. HIV transcription can also be autocatalytically increased by binding of the HIV tat protein to the tar region of the LTR. If two different proviruses are present in the same cell, transactivation through tat produced by either provirus could lead to synchronization of replication of both proviruses. There is also evidence that tat can be released from infected cells and be taken up into and transactivate tar sequences in other infected nearby cells (23). The tat/tar interaction might be thought of as a pheromone, or a specific mate recognition system that optimizes the temporal interactions between strains.

### The Lentivirus Gene Pool and Origins of Contemporary HIV Clades

At least 17 HIV clades have now been reported in humans: nine HIV-1 clades in the major grouping (A through I), three HIV-1 group O group "outlier" clades, and five HIV-2 clades. An additional three lentiviruses are known in nonhuman primate species (African green monkeys, mandrils, and Syke's monkeys). Thus the potential gene pool for primate lentivirus recombination is on the order of 20, e.g., 20 gag genes and 20 pol genes. The current HIV-1 clades may have arisen in part through past recombination between some of these genes.

Rates of transspecies infections with lentiviruses have not been measured. SIV has infected persons handling SIV-infected monkeys or virus cultures in the United States (24). Furthermore, phylogenetic data suggest that the human HIV-2 virus is almost certainly derived from SIV of sooty mangabeys in West Africa. Nucleotide sequence data suggest multiple sooty mangabey-to-human transspecies transmissions (25).

Viable recombinants between SIV and HIV ("SHIV" strains) have been genetically engineered in research laboratories for use in animal modeling experiments (26). No naturally occurring HIV-1/HIV-2 recombinants have been detected in human populations, but efforts to detect such strains have been very limited. Although SIV strains can productively infect humans and, therefore, might recombine with HIV-1, the lentiviruses of cats, horses, cows, and sheep have not productively infected humans and are unlikely to contribute to the pool of human lentivirus genetic elements.

### Barriers to HIV Recombination

Not all the theoretically possible combinations between HIV-1, HIV-2, and SIVs may give rise to recombinants in nature because of epidemiologic and biologic barriers.

### Segregation by Host Species

The frequency of transmission of viruses between primate species is not known. Intense surveillance for pox virus infections in equatorial Africa during the final decade of the smallpox eradication effort detected only 400 human monkeypox cases (27). Worldwide surveillance for herpes B virus infection, a common infection in nonhuman primates that is uniformly fatal in humans, has identified only 40 cases.

### Segregation by Geography

Although there are now perhaps 20 million HIV-infected persons worldwide, few (except in equatorial Africa) are likely to encounter partners infected with another clade. This is because, except in equatorial Africa, the HIV epidemic is genetically relatively homogeneous: B clade viruses predominate in Europe and North and South America, C clade viruses predominate in southern Africa and India, and E clade viruses predominate in Southeast Asia (28,29).

The current geographic distribution of clades and recombinants may not remain static. B clade and F clade mixing has begun in South America, and B/F recombinants have been detected. B clade and C clade mixing is occurring in South Africa, and E/A clade and C clade mixing is occurring in Asia, but new interclade recombinants have not yet been detected in these regions.

### Requirement for Multiple Infections in a Single Human

HIV-1 can superinfect persons who are chronically infected with HIV-2, but there is substantial heterotypic protection (30). Human infections with two or more HIV-1 clades have been recognized only rarely (31,32). While HIV-1-infected chimpanzees can be superinfected with a closely related strain under experimental conditions (33), it is still unclear if chronically HIV-1-infected humans are susceptible to superinfection by another HIV-1 strain through natural transmission. It may be that multiple infections with HIV-1 can occur in humans only when exposure to both viruses is near simultaneous.

### Requirement for Dual Infection of a Single Cell

Different variant HIV-1 strains can concurrently infect single cells in cell cultures in vitro, as can HIV-1 and HIV-2 (34). However, HIV downregulates its CD4 cell surface receptor, and dual infection of a single cell in vivo may require simultaneous attachment and penetration.

### Viral Structural Incompatibilities

The replication and synthesis of HIV virions is a complex process with molecular interactions at several levels: overlapping reading frames, RNA/RNA secondary structures, protein/RNA interactions, and intra- and intermolecular protein interactions. Recombination between two highly replication competent parent viruses might give rise to nothing but nonviable recombinant progeny because of incompatibilities in these molecular interactions.

### Consequences of Recombination for Prevention and Treatment of HIV Infections

The propensity of HIV strains to recombine has serious implications for epidemic control efforts.

### Epidemic Forecasting

If new strains—with new epidemiologic properties—can arise through HIV recombination as readily as new strains arise through reassortment in influenza A, then the long-term epidemiology of HIV may similarly be characterized by epidemic shifts and drifts. The emergence of the E/A recombinant clade in Southeast Asia may have simply been a chance event. However the fact that the E/A clade has spread much more rapidly than the B clade in this region raises concerns that some recombinants may emerge through natural selection based on their transmission efficiency (35).

### Antiviral Drug Resistance

Most HIV strains that are highly resistant to zidovudine (AZT) or to other antiviral drugs have multiple mutations, which act synergistically to confer the resistant phenotype to that drug. In vitro experiments in which single-mutant strains are grown in the presence of AZT show strong selection for recombinants bearing two or more resistance mutations (36). Multidrug resistant HIV-1 strains are likely to arise in patients treated with multiple drugs through recombination of variants that are resistant to single drugs (37). For example, crossover recombinants between strains singly resistant to a nucleoside analog and a protease inhibitor would be generated frequently in any cell coinfected with variants resistant to only one of these antiviral drugs.

Paradoxically, mutations in the reverse transcriptase that confer drug resistance might also serve to limit recombination. Mutations that confer AZT resistance increase the processivity of HIV-1 reverse transcription in vitro, but recombination rates of viruses bearing these mutations have not yet been studied (38).

### Vaccines

If new HIV strains are continually generated in nature through recombination, matching vaccines with prevalent genotypes in a particular geographic region may prove difficult. This may be less of a problem if small subunit vaccines are effective but may be more serious if complex vaccines constructed from antigens corresponding to two or more HIV gene products are needed. The need for a new influenza vaccine with each shift in the dominant epidemic influenza strain may be an instructive model. Another concern is that HIV genetic information from a vaccine might recombine with wild-type virus in vivo in an HIV-infected patient who was vaccinated, giving rise to new variants. Recombinant vaccinia strains containing HIV genes have already been tested in humans, and live attenuated and naked DNA HIV vaccines are being considered (39). Although in most instances it is unlikely that the new genetic information from the vaccine could give rise to more transmissible or more virulent strains, it is nonetheless possible. A relevant example is a recent epidemic in China, where gene sequences from the live attenuated oral polio vaccine were found to be stably recombined into the dominant virulent wild-type virus (40).

### Conclusion

Recombination may be an important fitness search strategy in the ongoing evolution of HIV. Many of the strains around the world appear to have arisen through recombination, and it is likely that recombination may be an important mechanism by which HIV evades drug or immune pressures. Future epidemiologic and clinical trials should examine the role of recombination in HIV evolution and adaptation, and computer models that simulate HIV mutation and recombination should be developed. The conceptual approach to HIV replication as a primitive sexual reproductive cycle might lead to new classes of interventions that block HIV evolution and adaptation.

### Acknowledgments

The author acknowledges valuable discussions with Drs. Francine McCutchan, Jean Carr, and Dan St. Louis; library search assistance from Ms. Pam Bell; and manuscript preparation assistance from Ms. Mary Hall.

Dr. Burke is interested in the molecular epidemiology of viral diseases. He conducted research on tropical infectious diseases, especially arthropod-borne viruses, hepatitis viruses, and HIV/AIDS. Dr. Burke is now professor of international health and director of the Center for Immunization Research at the Johns Hopkins University School of Hygiene and Public Health.

### References

1. Coffin JM. Genetic diversity and evolution of retroviruses. Curr Top Microbiol Immunol 1992; 176:143-64.
2. Hu W-S, Temin HM. Genetic consequences of packaging two RNA genomes in one retroviral particle: pseudodiploidy and high rate of genetic recombination. Proc Natl Acad Sci USA 1990;87:1556-60.
3. Robertson DL, Hahn BH, Sharp PM. Recombination in AIDS viruses. J Mol Evol 1995;40:249-59.
4. Temin HM. Sex and recombination in retroviruses. Trends Genet 1991;7:71-4.
5. Wain-Hobson S. The fastest genome evolution ever described: HIV variation in situ. Curr Opin Genet Dev 1993;3:878-83.
6. Holland J, Spindler K, Horodyski F, Grabau E, Nichol S, VandePol S. Rapid evolution of RNA genomes. Science 1982;215:1577-85.
7. Felsenstein J, Yokoyama S. The evolutionary advantage of recombination. II. Individual selection for recombination. Genetics 1976;83:845-59.
8. Kauffman SA. The origins of order. New York: Oxford University Press; 1993. p. 114-7.
9. Kirkegaard K, Baltimore D. The mechanism of RNA recombination in poliovirus. Cell 1986;47:433-43.
10. Zhang J, Temin HM. Retrovirus recombination depends on the length of sequence identity and is not error prone. J Virol 1994;68:2409-14.
11. Li WH, Tanimura M, Sharp PM. Rates and dates of divergence between AIDS virus nucleotide sequences. Mol Biol Evol 1988;5:313-30.
12. Jin MJ, Hui H, Robertson DL, Muller MC, Barre-Sinoussi F, Hirsch VM, et al. Mosaic genome structure of simian immunodeficiency virus from West African green monkeys. EMBO J 1994;13:2935-47.
13. Robertson DL, Sharp PM, McCutchan FE, Hahn BH. Recombination in HIV-1 [letter]. Nature 1995;9:374:124-6.
14. Louwagie J, Janssens W, Mascola J, Heyndrickx L, Hegerich P, van der Groen G, et al. Genetic diversity of the envelope glycoprotein from human immunodeficiency virus type 1 isolates of African origin. J Virol 1995;69:263-71.
15. McCutchan FE, Artenstein AW, Sanders-Buell E, Salminen MO, Carr JK, Mascola JR, et al. Diversity of the envelope glycoprotein among human immunodeficiency virus type 1 isolates of clade E from Asia and Africa. J Virol 1996;70:3331-8.
16. Sabino EC, Shpaer EG, Morgado MG, Korber BT, Diaz RS, Bongertz V, et al. Identification of human immunodeficiency virus type 1 envelope genes recombinant between subtypes B and F in two epidemiologically linked individuals from Brazil. J Virol 1994;68:6340-6.
17. Zhu T, Wang N, Carr A, Wolinsky S, Ho DD. Evidence of coinfection by multiple strains of human immunodeficiency virus type 1 subtype B in an acute seroconvertor. J Virol 1995;69:1324-7.
18. Diaz RS, Sabino EC, Mayer A, Mosley JW, Busch MP, the Transfusion Safety Study Group. Dual human immunodeficiency virus type 1 infection and recombination in a dually exposed transfusion recipient. J Virol 1995;69:3273-81.
19. Salminen MO, Carr JK, Robertson DL, Hegerich P, Gotte D, Koch C, et al. Evolution and probable transmission of inter-subtype recombinant HIV-1 in a Zambian couple. J Virol 1997;71:2647-55.
20. Paillart J-C, Skripkiin E, Ehresmann B, Ehresmann C, Marquet R. A loop-loop "kissing" complex is the essential part of the dimer linkage of genomic HIV-1 RNA. Proc Natl Acad Sci USA 1996;93:5572-7.
21. Huber HE, McCoy JM, Seehra JS, Richardson CC. Human immunodeficiency virus 1 reverse transcriptase: template binding, processivity, strand displacement synthesis, and template switching. J Biol Chem 1989;264:4669-78.
22. Fouchier RA, Meyaard L, Brouwer M, Hovenkamp E, Schuitemaker H. Broader tropism and higher cytopathicity for CD4+ T cells of a syncytium-inducing compared to a non-syncytium-inducing HIV-1 isolate as a mechanism for accelerated CD4+ T cell decline in vivo. Virology 1996;219:87-95.
23. Thomas CA, Dobkin J, Weinberger OK. Tat-mediated transcellular activation of HIV-1 long terminal repeat directed gene expression by HIV-1-infected peripheral blood mononuclear cells. J Immunol 1994;153:3831-9.
24. Khabbaz RF, Heneine W, George JR, Parekh B, Rowe T, Woods T, et al. Brief report: infection of a laboratory worker with simian immunodeficiency virus. N Engl J Med 1994;330:172-7.
25. Chen Z, Telfer P, Cettie A, Reed A, Zhang L, Ho D, et al. Genetic characterization of new West African simian immunodeficiency virus SIVsm: geographic clustering of household-derived SIV strains with human immunodeficiency virus type 2 subtypes and genetically diverse viruses from a single feral sooty mangabey troop. J Virol 1996;70:3617-27.
26. Luciw PA, Pratt-Lowe E, Shaw KE, Levy JA, Cheng-Mayer C. Persistent infection of rhesus macaques with T-cell-line-tropic and macrophage-tropic clones of simian/human immunodeficiency virus (SHIV). Proc Natl Acad Sci USA 1995;92:7490-4.
27. Jezek Z, Fenner F. Human monkeypox. In: Virology monographs. Basel (Switzerland): Karger; 1988. p. 17.
28. Hu DJ, Dondero TJ, Rayfield MA, George JR, Schochetman G, Jaffe HW, et al. The emerging genetic diversity of HIV. JAMA 1996;275:210-6.
29. Burke DS, McCutchan FE. Global distribution of HIV-1 clades. In: Devita VT, Hellman S, Rosenberg S, editors. AIDS: biology, diagnosis, treatment, and prevention. 4th ed. New York: Lippincott-Raven Publishers; 1997.
30. Marlink R, Kanki P, Thior I, Travers K, Eisen G, Siby T, et al. Reduced rate of disease development after HIV-2 infection as compared to HIV-1. Science 1994;265:1587-90.
31. Artenstein AW, VanCott TC, Mascola JR, Carr JK, Hegerich PA, Gaywee J, et al. Dual infection with human immunodeficiency virus type 1 of distinct envelope subtypes in humans. J Infect Dis 1995;171:805-10.
32. Heyndrickx L, Alary M, Janssens W, Davo N, van der Groen G. HIV-1 group O and group M dual infection in Benin [letter]. Lancet 1996;347:902-3.
33. Fultz PN, Srinivasan A, Greene CR, Butler D, Swenson RB, McClure Hm. Superinfection of a chimpanzee with a second strain of human immunodeficiency virus. J Virol 1987;61:4026-9.

34. Kim JH, McLinden RJ, Mosca JD, Burke DS, Boswell RN, Birx DL, et al. Transcriptional effects of superinfection in HIV chronically infected T cells: studies in dually infected clones. J Acquir Immune Defic Syndr Hum Retrovirol 1996;12:329-42.
35. Soto-Ramirez LE, Renjifo B, McLane MF, Marlink R, O'Hara C, Sutthent R, et al. HIV-1 Langerhans' cell tropism associated with heterosexual transmission of HIV. Science 1996;271:1291-3.
36. Kellam P, Larder BA. Retroviral recombination can lead to linkage of reverse transcriptase mutations that confer increased zidovudine resistance. J Virol 1995;69:669-74.
37. Gu Z, Gao Q, Faust EA, Wainberg MA. Possible involvement of cell fusion and viral recombination in generation of human immunodeficiency virus variants that display dual resistance to AZT and 3TC. J Gen Virol 1995;76:2601-5.
38. Caliendo AM, Savara A, An D, DeVore K, Kaplan JC, D'Aquila RT. Effects of zidovudine-selected human immunodeficiency virus type 1 reverse transcriptase amino acid substitutions on processive DNA synthesis and viral replication. J Virol 1996;70:2146-53.
39. Burke DS. Review: human trials of experimental HIV vaccines. AIDS 1995;9:S171-S80.
40. Zheng DP, Zhang LB, Fang ZY, Yang CF, Mulders M, Pallansch MA, et al. Distribution of wild type 1 poliovirus genotypes in China. J Infect Dis 1993;168:1361-7.
